# Cryoimmobilized anther analysis reveals new ultrastructural insights into *Rhynchospora* (Cyperaceae) asymmetrical microsporogenesis

**DOI:** 10.3389/fpls.2024.1518369

**Published:** 2025-01-21

**Authors:** Danilo M. Rocha, Ulla Neumann, Fernanda M. Nogueira, Georgios Tsipas, André L. L. Vanzela, André Marques

**Affiliations:** ^1^ Laboratório de Citogenética e Diversidade Vegetal, Departamento de Biologia Geral, Centro de Ciências Biológicas, Universidade Estadual de Londrina, Londrina, Paraná, Brazil; ^2^ Department of Chromosome Biology, Max Planck Institute for Plant Breeding Research, Cologne, Germany; ^3^ Departamento de Biologia, Faculdade de Filosofia Ciências e Letras de Ribeirão Preto (FFCLRP) – Universidade de São Paulo—USP, Ribeirão Preto, Brazil

**Keywords:** endoplasmic reticulum, high-pressure freezing, histochemistry, microspore mother cells, transmission electron microscopy

## Abstract

**Introduction:**

The Cyperaceae family is distinguished by holocentric chromosomes and a distinctive microsporogenesis process, which includes inverted meiosis, asymmetric tetrad formation, selective cell death, and the formation of pseudomonad pollen. Despite significant advances, the ultrastructural details of these processes remain poorly understood.

**Methods:**

This study provides a detailed analysis of microsporogenesis in *Rhynchospora pubera* using high-pressure freezing, freeze substitution, and transmission electron microscopy, significantly enhancing ultrastructural resolution.

**Results and discussion:**

Our findings reveal that intracellular organization differs from model species *Arabidopsis thaliana* and drives nuclear selection, with endoplasmic reticulum vesicles organizing meiotic spindles. Microtubules attach to centromeres located deep within holocentric chromosomes, while extensive cytoplasmic connections facilitate material exchange until callose deposition encloses meiocytes. Lipid distribution contributes to cell asymmetry, resulting in the characteristic asymmetric tetrads. Following meiosis, cytoskeletal elements coordinate nuclear migration and cell plate formation. Pseudomonads exhibit reconfigurations in the endomembrane system, particularly involving the endoplasmic reticulum, which supports functional cell differentiation. Complementary histochemical analyses corroborate these findings, providing insights into the cellular processes governing *Rhynchospora* microsporogenesis. These findings contribute to a deeper understanding of the developmental processes of Cyperaceae pollen, thereby facilitating future investigations of the underlying molecular mechanisms.

## Introduction

The Cyperaceae family, commonly known as sedges, exhibits several unique cytological features that distinguish it from other angiosperms. One of these features is the presence of holocentric chromosomes, which lack a localized centromere and instead have centromeric activity distributed along their length (Melters et al., 2012; Marques et al., 2015). Another distinctive feature is the simultaneous microgametogenesis occurring within each microsporocyte, resulting in the formation of a tetrad of nuclei within a wedge-shaped coenocytic cell (Kirpes et al., 1996; Furness and Rudall, 2000). Unlike the symmetric tetrads typically observed in angiosperms, sedges produce an asymmetric tetrad, with three nuclei displaced to the cell periphery, forming two distinct cellular environments: one functional and one degenerative (San Martin et al., 2013).

In most sedges, the degenerative nuclei are positioned in the narrower apical region of the microspore, facing the anther locule, whereas in *Rhynchospora* Vahl, they occupy the broader basal region, adjacent to the tapetum (Tanaka, 1941; Brown and Lemmon, 2000; San Martin et al., 2013). Despite numerous cytological studies, the biological basis for this difference remains unexplained. The asymmetrical tetrads formed in Cyperaceae consist of three smaller degenerative cells and one large functional cell that ultimately forms the mature pollen grain (Ranganath and Nagashree, 2000). The three smaller cells degenerate via programmed cell death, leading to the formation of a single functional pollen grain, which has earned the pollen of *Rhynchospora* the designation “pseudomonad” (Tanaka, 1941; Håkansson, 1954; Brown and Lemmon, 2000; Ranganath and Nagashree, 2000).

More recently, we have shown that the holocentric chromosomes in *Rhynchospora* undergo considerable restructuring in the transition from somatic to meiotic cells, with the absence of a longitudinal distribution of centromeric signals (Cabral et al., 2014; Marques et al., 2015; 2016). Remarkably, during early pollen development only the functional nucleus of the pseudomonad replicates its DNA and undergoes mitosis, while the three degenerative cells of pseudomonads, with unreplicated chromosomes initiate mitosis, leading to mitotic catastrophe and vacuolar cell death (Marques et al., 2016; Rocha et al., 2016). Despite these advances, many ultrastructural aspects of meiosis and pollen grain formation in Cyperaceae remain poorly understood.

Notably, the “cord of organelles,” a circular arrangement of mitochondria, proplastids, vacuoles, and other organelles surrounding chromosome complements during meiosis I and II, has been described in *Eleocharis* (Cyperaceae) and may play a role in the selection of the functional nucleus (Mariath et al., 2012). Another enigmatic feature is the presence of autophagic organelles in both degenerative and functional cells of *Rhynchospora*, suggesting a mechanism for the recycling of cytoplasmic components to support the functional cell (Rocha et al., 2016). Additionally, unusual endomembrane formations have been reported in sedge microspores and pollen grains (Mariath et al., 2012; San Martin et al., 2013; Rocha et al., 2020).

One challenge in studying pseudomonad development is the potential for artifacts arising from conventional chemical fixation, which can alter cell structures and lead to misinterpretation of biological processes (Li et al., 2017). Advances in cryoimmobilization and freeze-substitution techniques have improved the quality of cytological analyses in model plants by minimizing such artifacts (Yamamoto et al., 2003; Corral-Martínez et al., 2013; Mursalimov et al., 2022).

Here, we expanded our knowledge of Cyperaceae microsporogenesis using *Rhynchospora pubera* (Vahl) Boeckeler as a model. High-pressure freezing (HPF) and freeze-substitution (FS) for transmission electron microscopy (TEM) improved the ultrastructural resolution compared to previous studies. Our findings indicate that intracellular organization drives nuclear selection during meiosis, microtubules attach to centromeres deep within holocentric chromosomes, and cytoplasmic channels facilitate material exchange before callose obstruction. Additionally, pseudomonads exhibit reconfigurations in the endomembrane system involving the endoplasmic reticulum (ER), supporting functional cell differentiation. Complementary light microscopy provided further insights into holocentric chromosomes and pseudomonad development, laying a foundation for future research on pollen development in sedges.

## Results

### Organelle trafficking through cytoplasmic channels is observed in microspore mother cells

**Figure 1:**
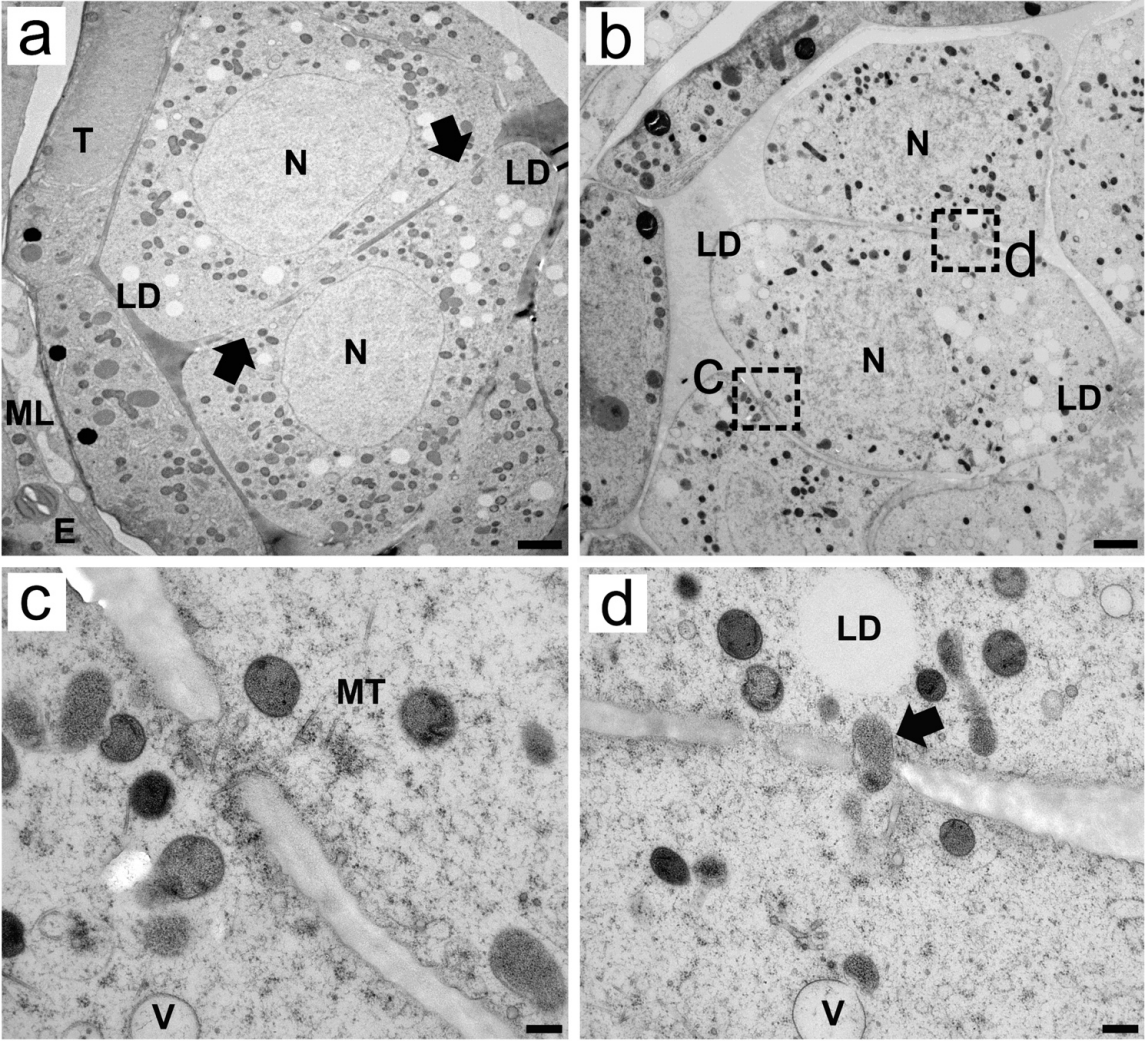
Transmission electron micrographs of transverse ultrathin sections of MMCs of *Rhynchospora pubera* prior to meiosis. **(A, B)** In young anthers, wedge-shaped MMCs prior to meiosis present large nuclei (N) with uncondensed chromatin. Their cytoplasm encompasses several small organelles, represented by electron-dense spheres and electron-translucent lipid droplets (LD) randomly scattered throughout the cytoplasm. Several cytoplasmic connections are present between adjacent MMCs [arrows in **(A)**]. The endothecium (E), middle layer (ML) and tapetum (T) are also depicted. Scale bar = 2 µm. The cytoplasmic connections between MMCs shown in B can be seen in detail in **(C, D)**. These connections are wide enough to contain microtubules (MT) **(C)** and entire organelles **(D)**. Small vacuoles are also observed nearby. Scale bar = 1 µm.

Analysis of high-resolution TEM images revealed that before meiosis, the microspore mother cells (MMCs) presented large nuclei that were centrally positioned, as well as numerous small organelles that were randomly dispersed in the cytoplasm (**Figure 1A**). Electron-transparent spheres that were not clearly delimited by a membrane appeared scattered throughout the cytoplasm. These structures represent lipid bodies or droplets, being electron-transparent due to cryosubstitution with acetone, and can be clearly detected in chemically fixed MMCs (**Supplementary Figure S1A**) and in isolated MMCs via brightfield and epifluorescence microscopy (see below). HPF-FS also enabled clear observation of cytoplasmic connections between MMCs. At early stages of pollen development, these connections are so large that they form a clear discontinuity between the cellular limits of the MMCs (**Figure 1A**). As development progresses, the space between MMCs becomes more evident, but the cytoplasmic connections remain present and are large enough for microtubules and large organelles to pass through (**Figures 1B–D**). These connections can also be observed in the TEM micrographs of chemically fixed MMCs (**Supplementary Figure S1A**).

### The cytoplasm during meiosis I shows organelle reorganization and a tilted spindle with bipolar attachment to holocentric chromosomes

**Figure 2:**
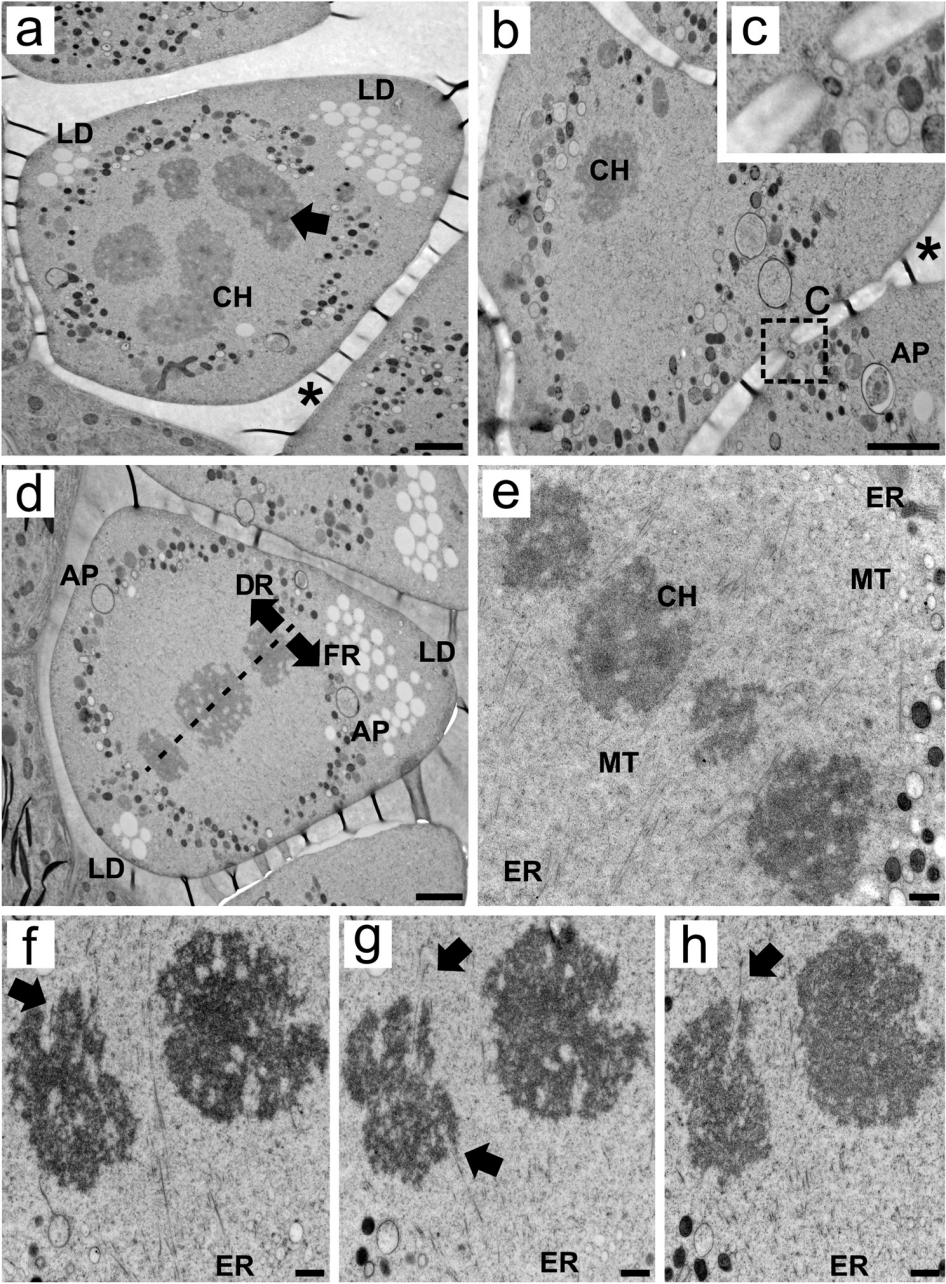
Transmission electron micrographs of transverse ultrathin sections of MMCs of *Rhynchospora pubera* during meiosis (I) **(A, B)** In prophase I, meiotic chromosomes are observed in the center of the cell. They contain small electron-dense areas (arrows). Organelles presented a circular organization that surrounded the chromosome set (CH). Lipid droplets (LD) are observed at two of the three angles of the wedge-shaped MMC but are particularly abundant in the opposite apical region. At this point, autophagosomes (AP) are also observed. Cytoplasmic connections large enough to allow the passage of organelles **(C)** still exist at this stage. Resin folds between MMCs appear as electron-dense threads (*), and are most likely favored by a difference in resin elasticity of neighboring subcellular domains (here: cell wall versus cytoplasm). Scale bar = 2 µm. **(D)** MMCs at metaphase I present chromosomes surrounded by organelles arranged in a circular matter, including autophagosomes (AP), with a predominance of lipid droplets (LD) in the apical region of the cell. The meiotic spindle is tilted so that one pole faces the basal degenerative region (DR) and the other faces the apical functional region (FR). Scale bar = 2 µm. **(E)** At meiosis I, the spindle microtubules are anchored by small endoplasmic reticulum (ER) vesicles at their poles. The chromosomes (CH) present bipolar attachment to the spindle, with microtubules (MT) interacting with chromosomes from both sides of the spindle. Scale bar = 500 nm. **(F–H)** Bipolar attachment of chromosomes to spindle microtubules was confirmed in serial ultrathin sections, which revealed that the microtubules entered the holocentric chromosome via small grooves on both sides (arrows). Meiotic chromosomes present irregular edges and intrachromosomal electron-translucent spaces. Scale bar = 500 nm.

During meiosis I, the cytoplasmic organization of MMCs shifted drastically as organelles, such as mitochondria and proplastids, appeared arranged circularly around the chromosome set. Numerous small lipid droplets were observed predominantly in the apical region, which is distant from the tapetum, but some were also located at the base angles of the MMC (**Figures 2A–D**). Cytoplasmic connections can still be observed during meiosis, allowing multiple organelles to pass through them (**Figures 2B, C**). Autophagosomes were also observed in the periphery of the cells during prophase I (**Figures 2B, D**).

The combination of HPF and FS improved the preservation of cellular structures and allowed a better observation of the relationships among spindle microtubules, endoplasmic reticulum (ER) cisternae, and chromosome sets during meiosis I. The microtubules were near small homogeneous ER cisternae at both poles of the spindle. These cisternae were arranged at specific points of the organelles that surround the chromosome set, one set near the basal region and the other near the apical region. Owing to this positioning, the spindle fibers were apparently tilted in relation to the greater axis of the MMCs. Spindle tilting directs one chromosome set to segregate to the basal, degenerative region to produce two of the three degenerative nuclei. Moreover, the other chromosomal set was directed to the opposite apical site (**Figures 2D, E**). This spindle organization was observed in other MMCs via TEM (**Supplementary Figures S2A, B**), as well as in serial sections imaged via light microscopy (**Supplementary Figure S3**).

During the condensation of chromosomes, electron-dense cores are often found at the center of the chromosomes (**Figure 2A**), suggesting the presence of a protein core. The structure of meiotic chromosomes in MMCs at metaphase I was very different from that observed in mitotic cells (**Supplementary Figures S1B, D**), as they presented intrachromosomal electron-translucent areas and irregular edges or small grooves, where microtubules are often inserted (**Figure 2D, E**). The spindle presents bipolar attachment to the chromosomes, as microtubules enter intrachromosomal spaces from opposite sides of the chromosomes, as can be observed in both single (**Figure 2E**) and serial sections (**Figures 2F–H**) at metaphase I. Few microtubules enter each groove at a given time.

### The cytoplasm shows a new organelle organization in meiosis II, while the functional nucleus is selected

**Figure 3:**
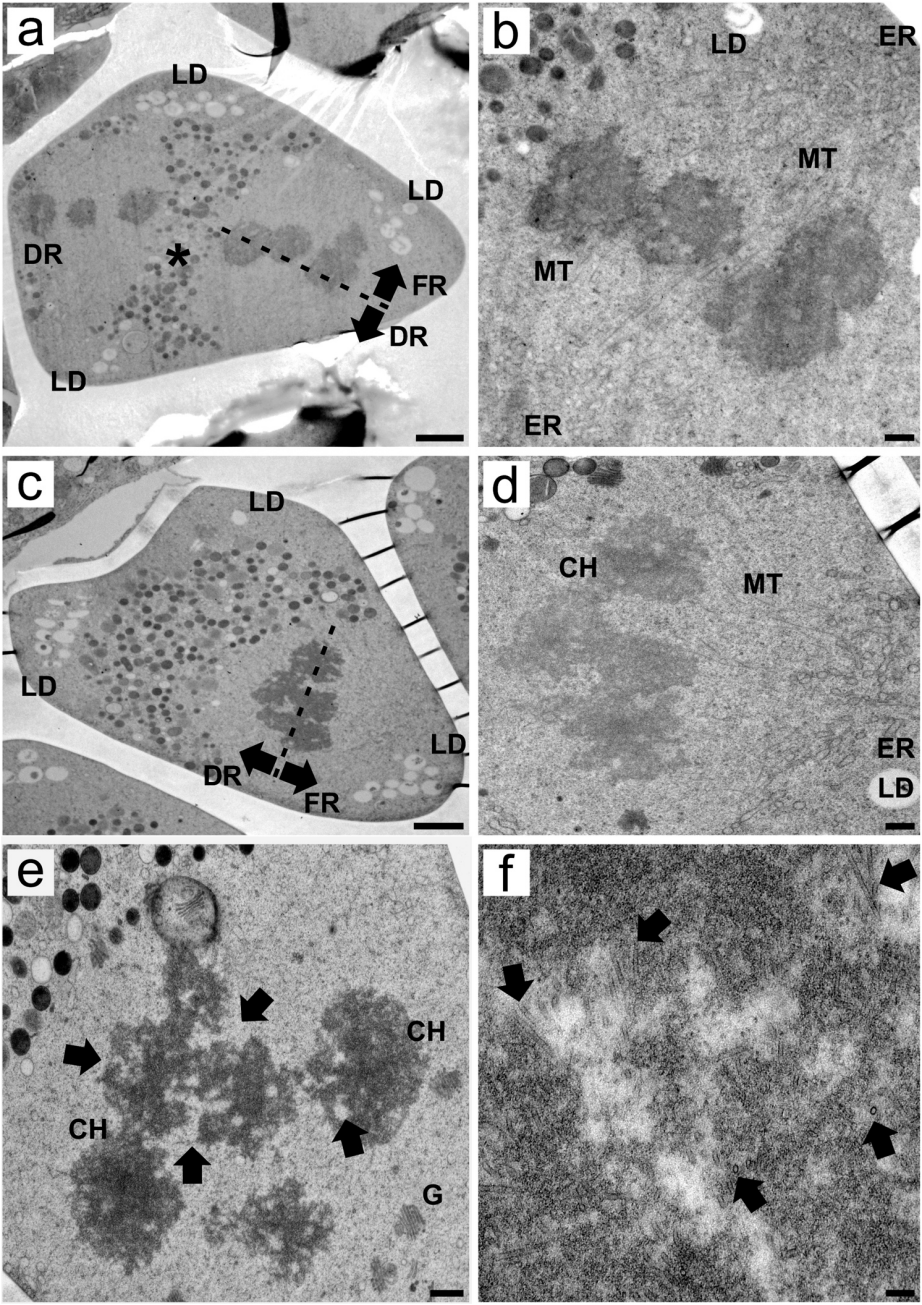
Transmission electron micrographs of transverse ultrathin sections of MMCs of *Rhynchospora pubera* during meiosis II. **(A)** At meiosis II, most of the MMC organelles are located in the middle portion of the cell between the two chromosome sets. Some lipid droplets (LD) are still observed in the apical region of MMCs, as well as at their base angles. The spindle is slightly tilted, with one pole oriented toward the basal, degenerative region (DR) and the other toward the apical, functional region (FR), adjacent to the lipid droplets. Scale bar = 2 µm. **(B)** Spindle microtubules (MT) in meiosis II enter the holocentric chromosome through small grooves. These microtubules are anchored by a small ER cisternae (ER) adjacent to lipid droplets (LD). Scale bar = 500 nm. **(C)** In another meiosis II MMC, the spindle was sectioned at an oblique angle, and the functional chromosome set segregated toward the apical region near the lipid droplets (LD). Scale bar = 2 µm. **(D)** A detail showing the association between the holocentric chromosomes (CH) and the spindle microtubule, the latter anchored by endoplasmic reticulum (ER) cisternae, in the vicinity of lipid droplets (LD). Scale bar = 500 nm. **(E)** Chromosomes (CH) during meiosis II present irregular edges and intrachromosomal electron-translucent spaces. Golgi bodies (G) are observed nearby. Scale bar = 500 nm. **(F)** Spindle microtubules penetrate deep inside the holocentric chromosome. In transverse sections of its inner portions, many microtubules (arrows) can be seen in different orientations. Scale bar = 0.1 µm.

During meiosis II, the cytoplasm presented a different configuration than what was observed in previous stages, with organelles accumulating in the median region of the MMC between the chromosome sets. Lipids accumulated at the angles of the cell (**Figure 3A**; **Supplementary Figure S2C**). Despite these differences, once again, the association between ER cisternae and spindle fibers was visible, where spindle microtubules were apparently anchored in small, homogeneous ER cisternae. The positioning of the ER cisternae was such that the spindle appeared to be slightly tilted, resulting in the segregation of one chromosome set, the functional set, to the apical region. The other set is segregated to the basal region of the MMC, becoming the third degenerative nucleus. Spindle tilting during meiosis II was also observed in other transmission electron micrographs (**Supplementary Figure S2D**) as well as in serial light microscopy sections (**Supplementary Figure S4**). Notably, the ER cisternae that anchor the spindle microtubules colocalize with lipid droplets (**Figures 3A, B**; **Supplementary Figure S2E, F**). In a transverse-oblique section of the spindle, the microtubules can once again be anchored to ER cisternae near apical lipid droplets, segregating the functional chromosome set to the apical region of the MMC (**Figures 3C, D**). Chromosomes in meiosis II resembled those in meiosis I, showing irregular edges and intrachromosomal electron-translucent spaces (**Figure 3E**), but no bipolar attachment was observed at this point. At higher magnifications, microtubules are inserted deep into the grooves and electron-translucent areas of the holocentric chromosome (**Figure 3F**).

### Cell plate formation separates functional and degenerative nuclei at the switch from telophase II to pollen mitosis I

**Figure 4:**
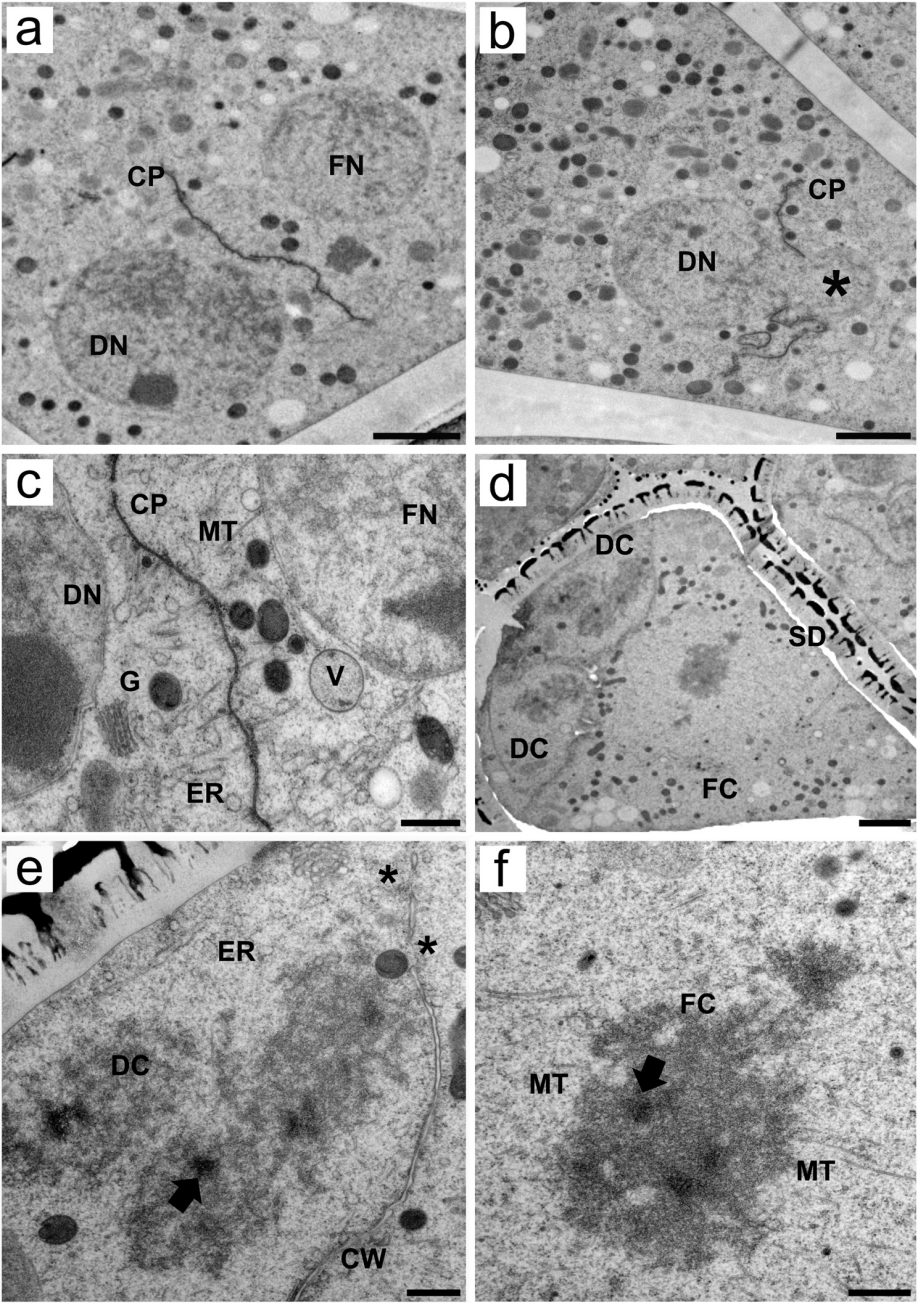
Transmission electron micrographs of transverse ultrathin sections of asymmetrical tetrads after meiosis revealed the formation of pseudomonads in *Rhynchospora pubera*. **(A)** After meiosis, degenerative (DN) and functional (FN) nuclei are formed, and cell plates (CP) begin to establish between them, while organelles are randomly scattered throughout the cytoplasm. Scale bar = 2 µm. **(B)** Snapshot of an incomplete cell plate (CP) forming around what appears to be a degenerative nucleus (DN) owing to its positioning. The formation of the cell plate occurs such that a large portion of the degenerative nucleus is “pouring into” the other side (*), which is the cytoplasmic region of the adjacent forming cell. Note once again the richness of organelles throughout the MMC cytoplasm. Scale bar = 2 µm. **(C)** High-magnification micrograph of a forming cell plate between a degenerative (DN) and a functional nucleus (FN). Numerous microtubules are attached to the cell plate. A large number of different organelles, such as endoplasmic reticulum (ER) cisternae, Golgi stacks (G) and vacuoles (V), can be observed. Scale bar = 500 nm. **(D)** As development progresses, the sporoderm (SD) becomes conspicuous, surrounding an asymmetrical tetrad, with two of the three smaller degenerative cells (DC) and the large functional cell depicted in this image (FC). Concomitantly, these cells also enter cell division, as condensation of chromosomes is observed in all cells (this would be pollen mitosis I in the functional cell). Scale bar = 2 µm. **(E, F)** As shown by the two high-magnification images of the tetrad shown in **(D)**, chromosome sets in degenerative **(E)** and functional **(F)** cells (DC and FC, respectively) are similar to the meiotic chromosomes seen previously, with irregular edges, intrachromosomal electron-translucent spaces and small electron-dense areas (arrows). However, microtubules (MT) can enter chromosomes through small grooves only in functional cells. Chromosomes also present bipolar attachment to the spindle. In degenerative cells, only the ER cisternae (ER) could be clearly observed. Note that the cell wall is still incomplete when cells enter division (*). Scale bar for **(E, F)** = 500 nm.

In telophase II, chromosomal sets are decondensed, and the nuclear envelope is reformed. Moreover, cytokinesis starts to occur via plate formation, separating degenerative cells from functional cells (**Figure 4A**). We obtained an image that shows a nucleus predominantly located in the basal, degenerative pole of the cell. A smaller portion of the same nucleus (see the * in **Figure 4B**), however, occupied the apical region. Cell plates formed between these two nuclear portions, causing constriction of the nucleus. During cell plate development, the separation of degenerative and functional cells is marked by an abundance of microtubules around the cell plate and organelles in the forming cells, including the endoplasmic reticulum, Golgi stacks (or bodies) and vesicles (**Figure 4C**).

**Figure 5:**
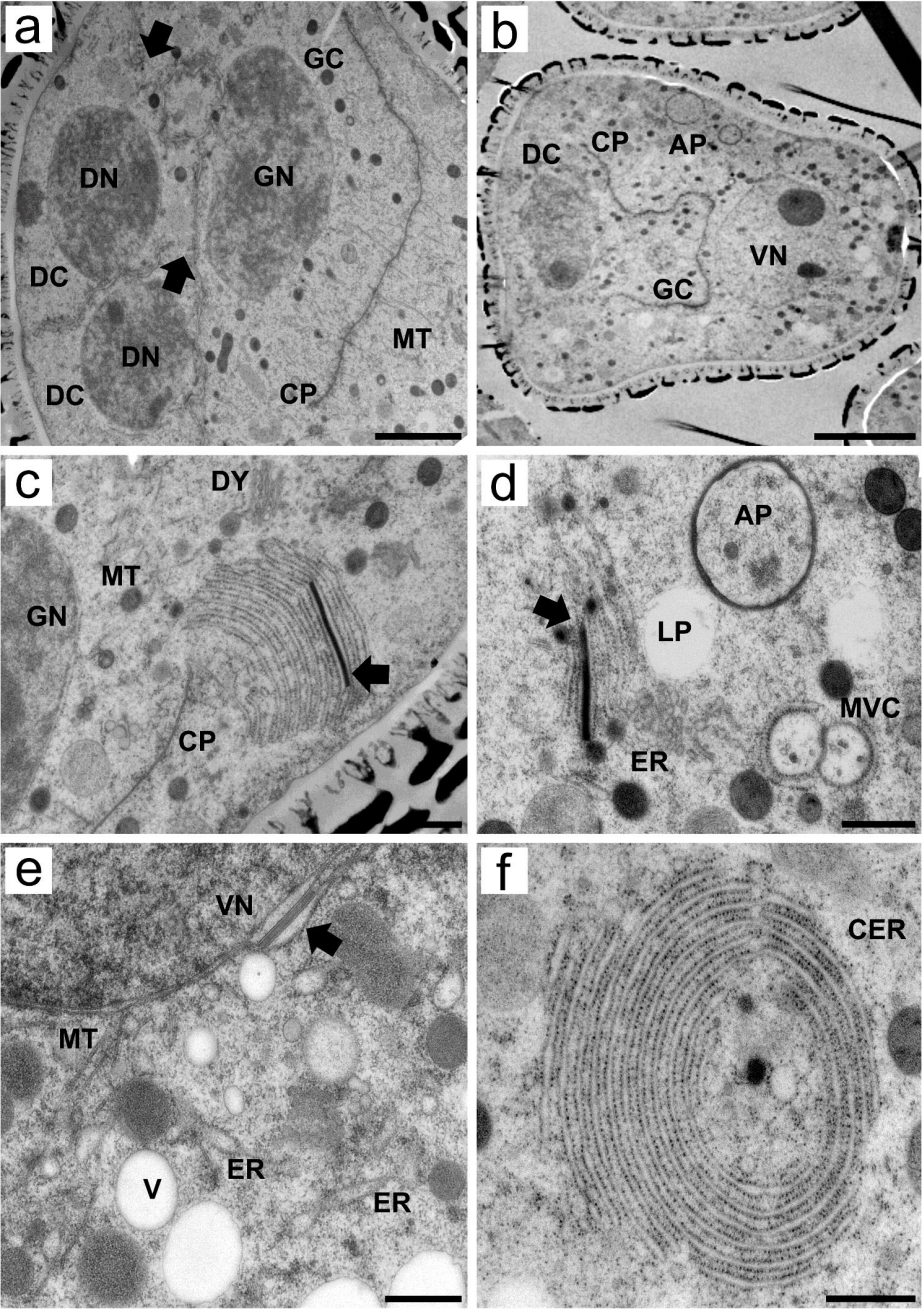
Transmission electron micrographs of transverse ultrathin sections showing microgametogenesis in *Rhynchospora pubera*. **(A)** Pollen mitosis I of the functional cell gives rise to the generative cell (CP), which is organized by the cell plate (CP) with associated microtubules (MT) and produces degenerative cells (DC). The plates between degenerative and functional cells (generative or vegetative) are still incomplete at this point, as demonstrated by the large cytoplasmic continuities between them (arrows). Due to sectioning, only two degenerative cells were visible. Scale bar = 2 µm. **(B)** As cell plate development progresses, the generative cell (CG) becomes more evident, adjacent to one of the three degenerative cells (DC). The vegetative cell occupies most of the pseudomonad volume, with the cytoplasm rich in organelles, including autophagosomes (AP), and a large nucleus with uncondensed chromatin and a prominent nucleolus. Scale bar = 5 µm. **(C)** High-magnification image of the vegetative cell cytoplasm showing a variety of organelles. As the cell plate (CP) is still separating the generative cell, with a generative nucleus (GN), mitochondria (M) and Golgi stacks **(G)** are located nearby. The endoplasmic reticulum (ER), which is arranged in parallel cisternae or short sheets, stands out for its proximity to the vegetative cell plasma membrane and for its peculiar electron-dense material between two of its cisternae (arrow). This rod-like material seems to be rigid in nature, as it shapes the nearby cisternae. Scale bar = 500 nm. **(D)** The electron-dense rod between the cisternae appears recurrently in the vegetative cell cytoplasm, near lipid droplets (LP) and autophagosomes (AP). Another interesting form of endoplasmic reticulum is observed, as some of its cisternae encompass small multivesicular bodies (MVC). Scale bar = 500 nm. **(E)** Details of the cytoplasm adjacent to a vegetative nucleus. Note the presence of an electron-dense rod between the nuclear envelope and an ER cisternae (arrow), both of which show a dilated lumen adjacent to the rod. Microtubules (MT), vacuoles (V) and other endoplasmic reticulum (ER) cisternae are observed nearby. Scale bar = 500 nm. **(F)** Cytoplasm of a vegetative cell with concentrically arranged endoplasmic reticulum (cER) cisternae surrounding cytoplasmic portions containing an electron-dense vesicle of unknown nature. Scale bar = 500 nm.

Shortly after telophase II, the functional cell of the newly formed pseudomonad undergoes asymmetric mitosis, i.e., pollen mitosis I (**Figures 4D–F**), which results in the formation of a vegetative nucleus and a generative cell (**Figure 5**). At this stage, the functional cell was larger than the degenerative cell and presented numerous organelles randomly distributed throughout the cytoplasm. Furthermore, the surface of the pseudomonad was adorn with a prominent exine composed of the outer tectum and inner baculae (**Figure 4D**). Even after entering mitosis, the cell plates from meiosis, between degenerative and functional cells, are still incomplete. Some organelles, such as the endoplasmic reticulum and mitochondria, are still present in degenerative cells (**Figure 4E**). Chromosomes in degenerative and functional cells exhibit ultrastructural features similar to those of meiotic chromosomes. The chromosome sizes, the presence of intrachromosomal, electron-translucent spaces, and patterns of microtubule insertions (seen clearly only in the functional cell chromosome), as well as the inner electron-dense regions in the chromosome, are comparable to those observed during meiosis (**Figures 4E**, **F**). The chromosomes of the functional cell clearly presented bipolar attachment to the spindle microtubules (**Figure 4F**).

### After pollen mitosis, microtubules drive cell plate formation, and vegetative cells exhibit distinctive features of endomembrane system organization

During telophase II and cytokinesis of pollen mitosis I, cell plate formation is driven, once again, by microtubules. Even at this point, the plates between degenerative and functional cells are incomplete. Generative cell formation occurs adjacent to degenerative cell formation (**Figure 5A**). As cytokinesis progresses, different cell types can be distinguished in developing pseudomonads. The degenerative cells, in the basal region, are the generative cells adjacent to them and the vegetative cells, which account for most of the volume of the pseudomonad. Degenerative cells are easily identified because they tend to have fewer organelles in their cytoplasm than vegetative cells do, which also present autophagosomes (**Figure 5B**).

The vegetative cell cytoplasm presented several peculiar ultrastructural features related to the endomembrane system. It presented extensive cisternae of the cortical ER adjacent to the plasma membrane (**Figure 5C**). One of the most intriguing ultrastructural features was the presence of an electron-dense, rod-shaped material, which was regularly deposited between the cisternae of the ER (**Figures 4C–E**). Electron-dense ER rods were detected as early as the stage of cell proliferation in the sporogenous tissue (**Supplementary Figure S1**), becoming both more frequent and obvious in the vegetative cells. This material appears to be rigid in shape, as it influences the shape of nearby cisternae (**Figure 5C**). In addition, the cisternae adjacent to the rod also appeared dilated (**Figures 5C, E**). This electron-dense rod-shaped material was also present between the nuclear envelope and adjacent ER cisternae (**Figure 5E**). The proximity and association of ER cisternae to multivesicular compartments, small vacuoles and autophagosomes (**Figures 5D, E**) were observed, as was the concentric circular ER arrangement (concentric ER) surrounding small portions of the cytoplasm, including other organelles (**Figure 5F**).

### Histochemical tests corroborate the presence of cytoplasmic connections and highlight the importance of lipids and actin filaments in the establishment of asymmetry

**Figure 6:**
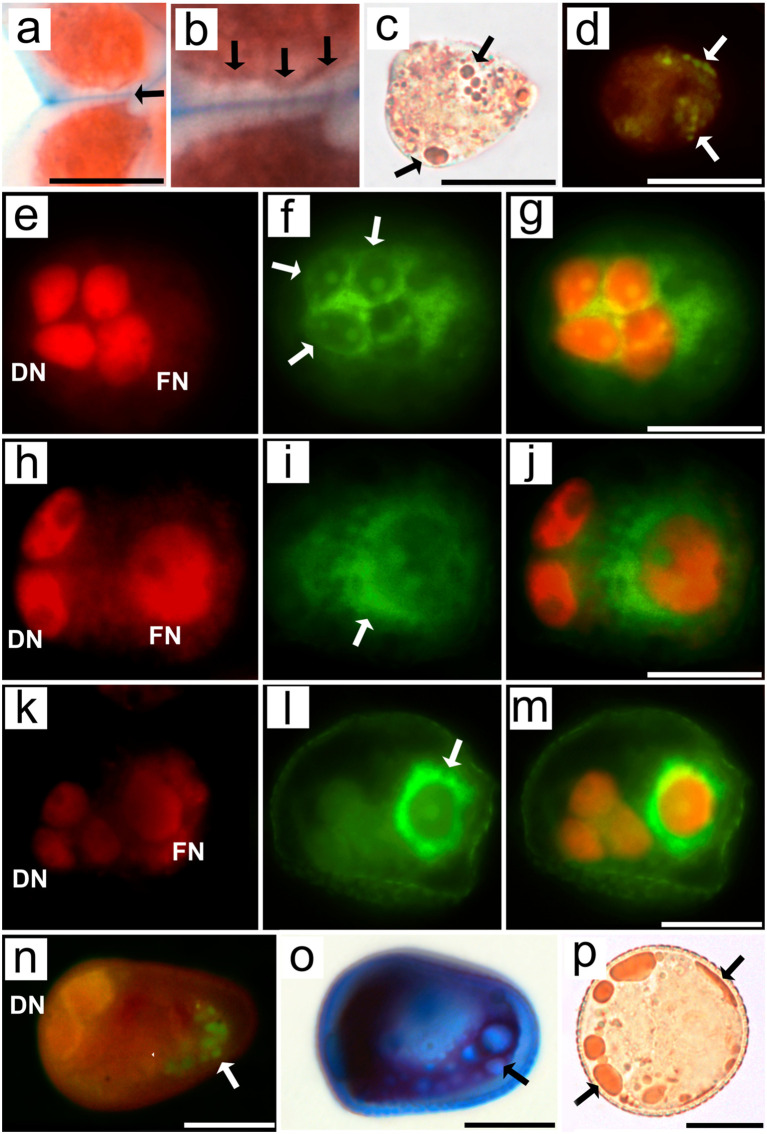
Light and epifluorescence micrographs of MMCs, asymmetrical tetrads and pseudomonads of *Rhynchospora pubera* after different histochemical treatments. **(A, B)** LR white sections of MMCs from *R. pubera* following double staining with Safranin and Astra Blue for cytoplasm (red) and primary cell wall staining (blue), while callose remained unstained. Several cytoplasmic threads (arrows) are observed between adjacent MMCs across their callose and primary walls. Scale bar in a = 5 µm. **(C)** Isolated MMCs after Sudan III staining, which revealed several lipid droplets of variable sizes (arrows), predominantly occurring in the narrower portion of the MMC. Scale bar = 5 µm. **(D)** Neutral red fluorescence in isolated MMCs confirmed the presence of lipid droplets (arrows) and their predominance in the apical region of the MMC. Scale bar = 5 µm. **(E–M)** Developmental series of MMCs from telophase II to the establishment of asymmetrical tetrads. Propidium iodide staining revealed early **(E)**, middle **(H)** and late telophase **(K)**, with degenerative nuclei in the basal region of the cell (left), becoming increasingly smaller, while the functional nucleus was dislocated to the other side of the cell. **(F)** Phalloidin-FITC fluorescence is present during early telophase II predominantly in the basal region of the cell (left), (I) becoming more present in the middle portion of the cell in mid telophase II, and later, in late telophase II **(I)**, strong phalloidin-FITC signals are observed in the functional region of the cell. The merged images confirmed that phalloidin-FITC signals were present around the degenerative nuclei in early telophase II **(G)**, between the degenerative nuclei and functional nucleus in mid-telophase II **(J)** and strongly concentrated around the functional nucleus in late telophase II **(M)**. Scale bars for G, J and M = 10 µm. **(N)** After a pseudomonad is established, lipids persist in the functional region, as indicated by neutral red fluorescence. Scale bar = 10 µm. **(O)** Alexander staining confirmed the deposition of noncytosolic material in the functional region. Scale bar = 10 µm. **(P)** As pollen grains are fully formed, lipids are present predominantly at the periphery of the vegetative cell (arrows). Scale bar = 10 µm.Supplementary information.

In addition to ultrastructural analyses via TEM, histochemical assays were used to test the relationship between cells and the asymmetric positioning of organelles, the cytoskeleton, and the cell plate during microsporogenesis and pollen grain development. The MMCs of *R. pubera* stained with safranine and Astra blue showed a large portion of callose (unstained) between the cell cytoplasm (red) and the middle lamella (blue). Multiple connections are observed between the MMCs, appearing as Safranin-stained lines across the callose wall (**Figures 6A, B**; **Supplementary Figures S5A, B**). These structures correspond to the cytoplasmic connections observed via TEM (**Figures 1**, **2**). The deposition of callose during meiosis was also confirmed by aniline blue staining (**Supplementary Figure S6**). The presence of lipid droplets in the narrower, apical part of early MMCs, as well as in their basal angles, was confirmed by staining with Sudan III (**Figure 6C**) and neutral red (**Figure 6D**).

Since actin filaments were not distinguishable in the TEM results, phalloidin-FITC was employed to visualize these cytoskeletal elements via fluorescence microscopy. Histochemical tests with phalloidin-FITC revealed a subtle accumulation of F-actin between the three similarly sized nuclei during telophase II. The slightly larger fourth nucleus (which would be the functional nucleus) was positioned toward the center of the cell and characterized by chromatin that is just subtly more decondensed. (**Figures 6E–G**). As asymmetry progresses, F-actin accumulates around the functional nucleus, separating it from the three degenerative nuclei (**Figures 6H–J**). As development continues, as indicated by the more conspicuous sporoderm, the functional nucleus is further away from the degenerative set and completely surrounded by F-actin (**Figures 6K−M**). Callose is also deposited between degenerative cells and functional cells (**Supplementary Figures S6M–O**).

Throughout pseudomonad development, the vacuolar system developed in general (**Supplementary Figure S5**), although some small lipid droplets were present in the apical region, as evidenced by neutral red staining (**Figure 6N**). Similarly, Alexander staining revealed an accumulation of noncytosolic material in the apical region at this stage of development (**Figure 6O**). The apical accumulation of noncytosolic material observed here is comparable to the accumulation of lipids observed via neutral red (**Figure 6N**). Mature pollen grains were recognized by their more rounded shape (**Figure 6P**; **Supplementary Figure S5H**) rather than the wedge-shaped pattern typical of previous stages. Additionally, mature pollen grains clearly accumulated lipids, which appeared as large drops. Interestingly, some lipid drops appear to accumulate very near the plasma membrane or even between the plasma membrane and the pollen wall (**Figure 6P**).

## Discussion

### MMC intracellular organization drives nuclear selection during meiosis

Cell asymmetry is a hallmark of microsporogenesis in *Rhynchospora* and other sedges (Brown and Lemmon, 2000; San Martin et al., 2009; Rocha et al., 2023 In most angiosperms, such as the model species *Arabidopsis thaliana*, organelles remain randomly dispersed throughout the cytoplasm before and during meiosis (Owen and Makaroff, 1995; Otegui and Staehelin, 2004). Pre-meiotic MMCs in sedges present a similar random organelle distribution, however, as the asymmetric meiosis progresses in sedges, specific organelle reorganizations are observed. During meiosis I, this configuration results in the chromosome set being surrounded by multiple organelles in a configuration that resembles a ring on images of thin sections. However, given that this structure is observed in every section during meiosis I, it can be inferred that it is an organelle shell rather than a ring. Subsequently, during meiosis II, organelles accumulate between chromosome sets. While mitochondria and proplastids are distributed uniformly throughout the organelle shell (meiosis I) and the organelle clusters between chromosome sets (meiosis II), ER cisternae, which are associated with spindle fibers, are preferentially localized at specific basal/apical points. Furthermore, lipid droplets are situated in a preferential manner at the apical aspect of the cell. These observations suggest that the cytoplasmic organization at this point plays a role in the asymmetric establishment process. Indeed, the formation of organelle shells indicates a high level of cytoskeletal and internal membrane organization prior to cell division. Similarly, this organelle arrangement has been observed in other plant families, including Malvaceae (Kudlicka and Rodkiewicz, 1990; Tchórzewska et al., 2008). The data from *Eleocharis sellowiana* (Mariath et al., 2012) indicate that this organelle arrangement may be associated with the development of cell asymmetry and that it may function as a selection mechanism for functional and degenerative nuclei.

A topic of ongoing debate in the field of asymmetric microsporogenesis in Cyperaceae is the mechanism by which a single nucleus, derived from the tetrad, is selected for the formation of the functional cell, while the remaining three nuclei undergo cell death (Ranganath and Nagashree, 2000; Marques et al., 2016; Rocha et al., 2016). It is currently unclear whether the functional nucleus is first selected and then relocated or becomes functional upon reaching the functional region of the MMC, potentially involving meiotic drive, as discussed by Furness and Rudall (2011). Studying mouse oocytes, Akera et al. (2017) observed that spindle asymmetry occurs prior to the initiation of meiosis and the formation of asymmetric cells. Meanwhile, during asymmetric cell division leading to stomata development in maize, certain proteins related to asymmetry establishment co-localize with the ER (Ashraf et al., 2023). Data on ER during microsporogenesis is, however, still scarce (Koç and De Storme, 2022). In this context, it appears that, in *R. pubera*, ER cisternae accompany spindle movement during meiosis, potentially tilting the spindle during meiosis I and II. If the spindle tilts, one chromosome set may be displaced toward the apical, functional region, which could potentially influence the selection process. Cabral et al. (2014) proposed that this selection of degenerative and functional nuclei occurs when homologous chromosomes are split during the final stages of meiosis II. Furthermore, our data on F-actin localization indicate that the actin cytoskeleton is involved in the processes of asymmetry and nuclear selection at this stage. Firstly, it is evident that F-actin is distributed in an uneven manner around degenerative and functional complements. Subsequently, F-actin accumulates in the vicinity of the functional nucleus, which may indicate that it is positioned in the center of the pseudomonad. The role of F-actin in nuclear positioning has also been observed in root cells (Ketelaar et al., 2002) as well as in microspores from other species, where nuclear positioning is necessary for achieving asymmetric pollen mitosis (Zonia et al., 1999).

In addition to the previously established correlation between the endoplasmic reticulum (ER) and meiotic spindles, our ultrastructural and histochemical data from *R. pubera* also indicate that lipid droplets are integral to the development of asymmetry. It is possible that lipid droplets serve a variety of functions in the process of cell differentiation and plant development. They provide the energy and carbon required for tissue growth, maintain cellular membranes, and facilitate the transport of enzymes and cell signals to organelles such as the endoplasmic reticulum and the Golgi apparatus (van der Schoot et al., 2011). Additionally, pollen grains accumulate due to the necessity of membrane formation for pollen tube growth (Ischebeck, 2016). The first sign of cell polarity, a necessary step for asymmetric division (Li, 2013), is seen in *R. pubera* when nucleus/chromosomes are located at the base of the cell, while lipid droplets occur predominantly at its apex. Chorlay et al. (2019) suggested that the phospholipid composition of ER membranes may influence the directionality of lipid droplets at the onset of cell division. Furthermore, the authors postulated that cytoskeletal-driven cytoplasmic movements may reposition ER cisternae and their associated lipid droplets, thereby creating an asymmetrical distribution. Nevertheless, the asymmetrical lipid accumulation in MMCs does not yet provide a comprehensive understanding of the opposing locations of the functional region observed in *Rhynchospor*a (apical) and other Cyperaceae (basal) in the pseudomonads (Brown and Lemmon, 2000; Rocha et al., 2018, 2023). Obtaining analogous data for other Cyperaceae genera would be beneficial for advancing this topic, as the positioning of degenerative cells in *Rhynchospora* differs from that observed in the majority of species within the family. Furthermore, the processes underlying polarity establishment in sedges during microsporogenesis and microgametogenesis appear to differ significantly from those observed in microgametogenesis in angiosperms: As seen in the model species *A. thaliana*, microspore polarity is established after cell vacuolation, while lipid accumulation apparently occurs only after pollen mitosis I (Owen and Makaroff, 1995). These differences highlight the diversity of mechanisms underlying cell asymmetry. As lipids serve a multitude of functions within cellular processes, elucidating their involvement in cell asymmetry is likely to remain a complex and challenging effort for years to come (Olzmann and Carvalho, 2019).

### Microtubules attach to centromeres located deep inside holocentric chromosomes during meiosis

The mitotic chromosomes of *R. pubera* exhibit centromere units inserted linearly through almost the full length of the longitudinal chromatid grooves (Marques et al., 2016). In contrast, in meiotic chromosomes, clusters of centromere units are present without a visible longitudinal groove (Marques et al., 2016). The differential condensation of meiotic chromosomes may provide an explanation for these observed differences (Rocha et al., 2016; Marques et al., 2016). The TEM data revealed the presence of varying degrees of condensation, as well as the emergence of electron-dense regions within the condensed chromatin in multiple micrographs. The electron-dense bodies exhibited regular positioning in different transverse sections, which resembled the electron-dense “chromatid cores” reported by Antonio et al. (1996) in *Chorthippus jucundus* (Orthoptera).

The irregular edges of the meiotic chromosomes of *R. pubera* were highlighted via TEM in comparison with those of the mitotic chromosomes. The irregular surface of the meiotic chromosome exhibits the presence of grooves and loops, which are characterized by widths of only a few nanometers. In contrast, the longitudinal mitotic grooves display widths that extend to the micrometer scale. This reduced size of the grooves could explain the difficulty in observing them in meiotic chromosomes via light microscopy, as previously noted by Marques et al. (2016). The attachment of microtubules to the opposing grooves during meiosis I (bipolar attachment) is essential for the separation of sister chromatids during inverted meiosis, as documented by Cabral et al. (2014). This would result in the segregation of homologous chromosomes at meiosis II. It would be highly beneficial to extend these meiotic investigations to additional Cyperaceae species to ascertain whether this chromatin configuration is present in taxa where no repeat-based holocentromeres are documented.

### Cytoplasmic channels permit the exchange of large quantities of materials before callose obstruction

A striking feature of the MMCs of *R. pubera* was the presence of multiple cell−cell bridges before and throughout meiosis, which sometimes contained entire organelles of different sizes. Plasmodesmata are common connections maintained by ER cisternae that obstruct cell plate formation at some sites (typically 0.1–2 μm wide), enabling trafficking between adjacent cells (Ghoshroy et al., 1997; Miras et al., 2022). These connections have previously been observed in MMCs of *A. thaliana* (Otegui and Staehelin, 2004). Primary plasmodesmata can be complex and diverse in tissues undergoing intense differentiation, allowing for the diffusion of large compounds (see Miras et al., 2022). However, the size and absence of a desmotubule, such as that shown here and by San Martin et al. (2009), makes this structure different from typical plasmodesmata. It would be more reasonable to explain them as large cytoplasmic channels or bridges, which allow the exchange of large portions of cytoplasmic material, including entire organelles and microtubules. This high number of cell−cell bridges observed in early meiosis in *R. pubera* may play an important role in maintaining synchrony for microsporogenesis. Eventually, large channels such as these can even allow the passage of a nucleus or nuclear portions between cells, which is often referred to as cytomixis (Wang et al., 2004; Mursalimov and Deineko, 2018).

The control of callose deposition is a determinant of maintaining cell connections (Sager and Lee, 2018). Our findings indicate that communication between MMCs prior to meiosis is maintained, but as of prophase I, it decreases as meiosis progresses, coinciding with the time of callose deposition. In many angiosperms, callose accumulation occurs during microsporogenesis until four microspores are released, but in Cyperaceae, this exact same process does not occur. Instead, callose is deposited between individual pseudomonads, isolating them. Nevertheless, its role could be to isolate cells/microspores once meiotic synchronicity is established, not only in sedges but also in angiosperms in general. The plasmodesmatal channel components and genetic factors that control plasmodesmata are still unexplored (Sager and Lee, 2018). Our data based on high-resolution images in *R. pubera* suggest that these cell−cell bridges could provide significant insights into MMC synchrony (Heslop-Harrison, 1966) and, perhaps, into possible mechanisms that lead to cell asymmetry. Another important cytoplasmic connection observed in *R. pubera* was between degenerative and functional cells. These cell−cell bridges probably arose from incomplete cell plate formation, as it does in MMCs (Risueño et al., 1969). The cytoplasmic connections between degenerative cells could be related to their common mechanisms of programmed cell death (Rocha et al., 2016). This implies that cellular signals that direct functionality or programmed cell death are triggered after complete formation of the cell plate, which coincides with the timing of cell death in pseudomonads, which occurs after pollen mitosis I (Rocha et al., 2018). The cytoplasmic connections between degenerative and functional cells are closed due to callose deposition, ensuring proper generative and vegetative cell development (also observed in other instances in *R. pubera*, as well as in *E. sellowiana*, Rocha et al., 2018). Investigating the possible occurrence of cytomixis through these cytoplasmic channels in MMCs and pseudomonads in Cyperaceae would be highly interesting, as traveling holocentric fragments could be more easily incorporated in this case.

### Pseudomonads have a versatile endomembrane system

The vegetative cells of pseudomonads are rich in organelles, as observed in the vegetative cells of other plant species’ pollen grains (Otegui and Staehelin, 2004). In *R. pubera* pseudomonads prepared by HPF-FS, the well-developed endoplasmic reticulum network, including diverse unusual formations, was particularly striking. Among these, the cortical ER is one of them. This structure, which has been reported in somatic cells and mature pollen of several other plant species, might be related to calcium signaling, cell secretion, and anchoring points for intracellular movements (Otegui and Staehelin, 2004; Rocha et al., 2020; Kriechbaumer and Brandizzi, 2020). By using cryoimmobilization, a more precise observation of the part of the endomembrane system associated with autophagy could be achieved. Chemically fixed pseudomonads of *Rhynchospora* (Mariath et al., 2012; San Martin et al., 2013; Rocha et al., 2020) have already provided evidence of these structures, but the occurrence and structure of autophagic vesicles close to the concentric and linear cisternae of the ER, as well as the lipid droplets, have become more evident. Similar autophagy-related features have been observed in microspores of other plant species (Tchórzewska et al., 2008), megaspore mother cells of *Eleocharis* (Rocha et al., 2015) and many other different cells in other plant species (Maeda and Miyake, 1997; Suzuki et al., 2001). The autophagy of the ER or microER-phagy (Wilkinson, 2019) could be important for cytoplasmic turnover and reorganization in MMCs, microspores and vegetative cells, explaining their concentric formation and association with organelles of the endocytic pathway (endosomes in the form of multivesicular bodies and lytic vacuoles). Autophagic bodies and concentric ER formations are also seen in the model species *A. thaliana*, indicating that, despite the many differences in microsporogenesis and microgametogenesis described earlier, cytoplasmic reorganization processes remain conserved between sedges and angiosperms (Otegui and Staehelin, 2004), which highlight the importance of these events.

Electron-dense rods between ER cisternae are also notable features of the endomembrane system. This structure appears repeatedly in MMCs before meiosis and is present in large quantities in vegetative cells. This phenomenon has never been observed in Cyperaceae, and we have also not found it in reports referring to MMCs, microspores, and pollen grains from other plant cells. Records of electron-dense material accumulation between ER cisternae during sieve element differentiation are attributed to metabolic activity, enzyme accumulation or P-protein accumulation (Esau and Gill, 1971, 1972). However, these rods are clearly different from intercisternal ER rods because they are granular in nature and appear throughout the entire ER. The structures observed here might serve as scaffolds for the stacking of ER cisternae in very active tissues and could avoid microER-phagy in some cytoplasmic regions, as their rigidity could prevent the circularization of the ER associated with it.

## Conclusion

Cryoimmobilization significantly improved the quality of the ultrastructural images obtained during microsporogenesis in the model species *R. pubera*. This enhancement was evident, as different cytoplasmic rearrangements could be characterized, including a complex composed of organelles, lipid droplets and meiotic spindles that likely contribute to the displacement of the functional chromosome set to the apical region of the pseudomonad and the selection of the functional meiotic product. It was also possible to observe nanometer-scale grooves in the meiotic chromosomes where microtubules penetrate deeply, and bipolar attachment to holocentric chromosomes in meiosis I was confirmed, corroborating the occurrence of inverted meiosis in *R. pubera*. The records of cytoplasmic connections in MMCs have also improved greatly with cryoimmobilization, and these connections can be characterized as large cytoplasmic bridges allowing the passage of multiple organelles and microtubules. Investigating the possible occurrence of cytomixis in Cyperaceae through these channels would be interesting, as holocentric fragments could be incorporated by meiocytes in these events. Another interesting observation was the ultrastructural features of vegetative cells, which are related to ER formation associated with autophagy and yet uncharacterized intercisternal rods between ER cisternae. While considerable insight has been gained into the microsporogenesis of *Rhynchospora* through the use of cryoimmobilization, further investigation into this process in other sedges, particularly those exhibiting disparate pseudomonad organizations, such as *Carex* and *Eleocharis*, is essential to enhance our understanding of this phenomenon.

## Materials and methods

### Plant material

Individuals of *R. pubera* were collected in the field at Recife, Pernambuco, Brazil, and maintained in the greenhouses of the Laboratory of Cytogenetics and Plant Diversity at the State University of Londrina, Brazil, and the Max Planck Institute for Plant Breeding Research, Germany. Vouchers were deposited in the FUEL herbarium under voucher number 55374.

### Light microscopy

For light microscopy analysis, anthers and inflorescences were subjected to different treatments: i) For general observations, inflorescences were fixed in 4% formaldehyde solution (obtained from paraformaldehyde) in 1x PBS for four hours, washed in the same buffer, dehydrated in a graded ethanol series (10–100%), infiltrated with and embedded in LR white resin. Thin sections (5 µm) were obtained via a Leica^®^ RM2245 microtome, and the sections were stained with Safranin and Astra Blue (modified from Kraus et al., 1998). ii) For F-actin detection, anthers were fixed in 4% formaldehyde in 1x PBS as described previously and washed in the same buffer, followed by cell wall digestion in a 4% cellulase, 20% pectinase, and 2% hemicellulase (v/w/v) enzyme solution for two hours at 37°C. Afterward, the anthers were dissected in 1x PBS, mounted between slides and coverslips and frozen in liquid nitrogen. Coverslips were removed after freezing to open the cells by freeze-fracturing. The slides were then treated with a 5% phalloidin-fluorescein isothiocyanate (FITC) solution, in which phalloidin binds strongly to F-actin, and subsequently stained with a 10 μg/mL solution of propidium iodide in distilled water for DNA/nuclear staining. The slides were then mounted with antifade solution (25 μl) composed of DABCO [1,4-diaza-bicyclo(2.2.2)-octane (2.3%)], 20 mM Tris–HCl pH 8 (2%), 2.5 mmol l^–1^ MgCl2 (4%), and glycerol (90%) in distilled water. iii) To observe viable cytoplasm, fresh anthers were directly dissected with 1% Alexander stain (Alexander, 1969) and prepared as semipermanent slides. iv) For total lipid detection, fresh anthers were directly dissected in Sudan III (Johansen, 1940). Images were acquired using a Leica DM5500B microscope equipped with a Leica DFC300FX camera. Additionally, anthers were directly dissected with the lipid fluorochrome neutral red (Kirk, 1970). Widefield fluorescence images were recorded using a Leica DM5500B microscope equipped with a Leica DFC300FX camera.

### Transmission electron microscopy

The samples for TEM analysis were processed via high-pressure freezing, freeze-substitution and room-temperature resin embedding. Anthers at different stages of development were harvested from plants grown in the greenhouse (16 h light, 26–27°C, 70–80% humidity). Depending on the size of the anther, whole anthers or anther fragments were loaded into aluminum specimen carriers (outer diameter 3 mm) with 300 µm deep cavities (Leica Microsystems GmbH), prefilled with 1-hexadecene, capped with a second specimen carrier (flat side toward the sample) and immediately frozen in a Leica EM HPM 100 high-pressure freezer (Leica Microsystems GmbH). Freeze-substitution in either 2% or 2.5% osmium tetroxide in acetone was performed in a Leica EM AFS2 freeze substitution device (Leica Microsystems GmbH), and the temperature was gradually increased over 72 hours from -85°C to 4°C under constant agitation (Reipert et al., 2018). After agitation for an additional one to two hours at room temperature in the freeze-substitution medium, the samples were rinsed in acetone, carefully removed from the aluminum sample carriers and gradually infiltrated into Araldite 502/Embed 812 resin (Science Services, Munich, Germany) over 5 days. Infiltration into pure resin was facilitated by ultracentrifugation (McDonald, 2014); resin polymerization was performed in flat embedding molds at 60°C for 48 h. Ultrathin sections (≈70 nm) were cut and collected on Formvar-coated nickel slot grids (Moran and Rowley, 1987). Depending on the target structure/organelle, different staining methods were applied: 0.1% potassium permanganate in 0.1 N H_2_SO_4_ for one minute alone (Sawaguchi et al., 2001) or followed by 0.5% uranyl acetate in water (w/v) for 10 minutes and/or lead citrate for 15 min. When long series of ultrathin sections distributed onto numerous grids were stained, single potassium permanganate contrast was often applied to minimize the risk of losing individual grids during contrast. Sections were examined with a Hitachi H-7650 or HT7800 transmission electron microscope (operating at 100 kV) equipped with an AMT XR41-M or EMSIS Xarosa digital camera, respectively. For conventional chemical fixation, samples were processed as described in Koskela et al. (2018), with the exception that 0.1 M instead of 0.05 M sodium cacodylate buffer was used and that glycine was omitted from the washing steps between primary and secondary fixation.

## Data Availability

The original contributions presented in the study are included in the article/**Supplementary Material**. Further inquiries can be directed to the corresponding authors.

## Supplementary material

Supplementary Figure 1 Transmission electron micrographs of ultrathin sections of different samples of *R. pubera* processed by chemical fixation. **(A)** Transversal section of an anther locule, showing middle layer (ML), tapetum (T) and an adjacent pre-meiotic microspore mother cell. The latter shows a prominent nucleus and organelles scattered throughout the cytoplasm, including electron-dense lipid droplets, as well as large cytoplasmic connections to adjacent microspore mother cells **(B)**. Scale bar in A = 2 µm. **(C)** A mitotic chromosome (CH) in root cells, in a longitudinal section. This rod-shaped chromosome presents regular edges, where microtubules (MT) attach to. Scale bar = 500 nm. **(D)** A transversal section of the sporogenous tissue in a young anther shows mitotic chromosomes (CH) with electron-dense cores (Arrow). A rod-shaped electron-dense material is also seen in the cytoplasm (*). Scale bar = 2 µm. **(E)** Detail of the mitotic chromosome in **(D)** shows its regular edges and electron-dense central region. Scale bar = 500 nm.

Supplementary Figure 2 Transmission electron micrographs of transversal ultrathin sections of microspore mother cells of *R. pubera* during meiosis. **(A)** At metaphase I, chromosomes are surrounded by a circular arrangement of organelles, which include autophagosomes (AP). Lipids droplets (LD) are located mainly in the apical region of the cell. Due to the tilted arrangement of the meiotic spindle, one of its poles faces the basal, degenerative region (DR) and the other faces the apical, functional region (FR). Scale bar = 2 µm. **(B)** During meiosis I, the spindle microtubules (MT) appear to be anchored by small endoplasmic reticulum cisternae (ER) at the spindle poles. Chromosomes (CH), autophagosomes (AP) and lipid droplets (LD) are also seen. Scale bar = 2 µm. **(C)** At prophase II, two chromosome sets (CH) are seen in opposite sides of the cell, with numerous organelles between them. Scale bar = 2 µm. **(D)** At metaphase II, the apical spindle presents one pole oriented towards the basal, degenerative region (DR) and the other to the apical, functional region (FR), adjacent to the apical lipid droplets (LD). These droplets are also seen in the angles of the cell. Scale bar = 2 µm. **(E)** Different cells in meiosis II are seen, with lipid droplets accumulating in their respective apical regions, as well as at their base angles. Scale bar = 10 µm. Micrograph of the boxed region in **(F)** shows that chromosome segregation in the basal region of the cell is also organized by spindle microtubules (MT) anchored to endoplasmic reticulum cisternae (ER) adjacent to lipid droplets (LD). The spindle microtubules penetrate deep inside the holocentric chromosome (arrows). Scale bar = 500nm.

Supplementary Figure 3 Light micrographs of serial transversal sections of an *R. pubera* anther in meiosis I. Three serial sections of the same locule are represented in **(A–E, G–L)**. In **(A, D, G, J)**, the orientation of the spindle (white dotted line) against the major axis of the cell (black dotted line) is represented. In the other images, the same cells are also seen in different sections (black arrows). Asterisks in **(B, C)** show two chromosome sets in different section planes, one to the left apical side and the other to the right basal side. Scale bar = 10 µm.

Supplementary Figure 4 Light micrographs of serial transversal sections of an *R. pubera* anther in meiosis II. Three serial sections of the same locule are represented in **(A–E, G–I)**. Cells at metaphase II are seen with tilted spindles (black arrows). Scale bar = 10 µm. Asterisks in **(D, E)** show two chromosome sets in different section planes, one to the left apical side and the other to the right basal side. Figure **(H)** shows a possible anaphase II with the same configuration (*). Scale bar = 10 µm.

Supplementary Figure 5 Light micrographs of longitudinal sections showing anther development in *R. pubera.* **(A)** Microspore mother cells present cytoplasm well stained by safranin, while pectic cell walls are stained by astra blue (arrow). Callose walls are unstained. **(B)** Detail of **(A)** showing cytoplasmic connections between adjacent microspore mother cells as thin threads crossing the common cell wall (arrows). The same is seen for other microspore mother cells **(C–F)**. Scale bars in a, c and e = 10 µm. **(G)** After meiosis and pollen mitosis I, vegetative cells present large vacuoles (*). Remains of the degenerative nuclei are seen in the basal region of the pseudomonad (arrowhead). In the anther wall, the endothecium (EN) and remains of the tapetum are also seen (T). Scale bar = 10 µm. **(H)** Mature pollen grains of *R. pubera* present densely stained cytoplasm, with a vegetative cell, with a vegetative nucleus (VN), and two sperm cells adjacent to it (arrows). Scale bar = 10 µm.

Supplementary Figure 5 Epifluorescence micrographs of longitudinal sections of *R. pubera* anthers at different stages of development. Development series of MMCs from before meiosis to the establishment of asymmetrical tetrads following double staining with propidium iodide **(A, D, G, J, M)**, Aniline Blue **(B, E, H, K, M)**, which stains callose and merged images of propidium iodide and Aniline Blue **(C, F, I, L, O)**. **(A–C)** Before meiosis, no callose deposition can be seen. **(D–F)** During prophase I, callose deposition becomes noticeable. Callose is still seen during prophase II **(G–I)** and metaphase II **(J–L)**. At telophase II **(M–O)**, callose is seen between microspore mother cells, as well as between the nuclei arising from meiosis (arrows in N). Scale bar = 10 µm.
