# Supplementary material for: Cryoimmobilized anther analysis reveals new ultrastructural insights into Rhynchospora (Cyperaceae) asymmetrical microsporogenesis
Source: Front Plant Sci. 2025 Jan 21;15:1518369. doi: 10.3389/fpls.2024.1518369 (PMC11790663; doi:10.3389/fpls.2024.1518369)
Supplement: Supplementary Figure 1 — Transmission electron micrographs of ultrathin sections of different samples of R. pubera processed by chemical fixation. (A) Transversal section of an anther locule, showing middle layer (ML), tapetum (T) and an adjacent pre-meiotic microspore mother cell. The latter shows a prominent nucleus and organelles scattered throughout the cytoplasm, including electron-dense lipid droplets, as well as large cytoplasmic connections to adjacent microspore mother cells (B). Scale bar in A = 2 µm. (C) A mitotic chromosome (CH) in root cells, in a longitudinal section. This rod-shaped chromosome presents regular edges, where microtubules (MT) attach to. Scale bar = 500 nm. (D) A transversal section of the sporogenous tissue in a young anther shows mitotic chromosomes (CH) with electron-dense cores (Arrow). A rod-shaped electron-dense material is also seen in the cytoplasm (*). Scale bar = 2 µm. (E) Detail of the mitotic chromosome in (D) shows its regular edges and electron-dense central region. Scale bar = 500 nm. [file DataSheet1.pdf]

Supplementary information

Cryoimmobilized anther analysis reveals new ultrastructural insights into *Rhynchospora* (Cyperaceae) asymmetrical microsporogenesis

Danilo M. Rocha[1,#], Ulla Neumann[2,#], Fernanda M. Nogueira[3], Georgios Tsipas[2], André L. L. Vanzela[1,*], André Marques[2,*]

[1]*Laboratório de Citogenética e Diversidade Vegetal, Departamento de Biologia Geral, Centro de Ciências Biológicas, Universidade Estadual de Londrina, Londrina, 86097-570, Paraná, Brazil.*

[2]*Department of Chromosome Biology, Max Planck Institute for Plant Breeding Research, Carl-von-Linné-Weg 10, 50829 Cologne, Germany*

[3]*Laboratório de Biologia Molecular de Plantas, Departamento de Biologia, Faculdade de Filosofia Ciências e Letras de Ribeirão Preto (FFCLRP) – Universidade de São Paulo—USP, Ribeirão Preto, 14040-901, Brazil*

#These authors contributed equally to this work.

*Correspondence to andrevanzela@uel.br and amarques@mpipz.mpg.de

Danilo M. Rocha: https://orcid.org/0000-0003-0059-7962

Ulla Neumann: https://orcid.org/0000-0001-9200-4209

Fernanda M. Nogueira: https://orcid.org/0000-0003-1830-4929

Georgios Tsipas: https://orcid.org/0000-0002-1995-0327

André L. L. Vanzela: https://orcid.org/0000-0002-2442-2211

André Marques: https://orcid.org/0000-0002-9567-2576

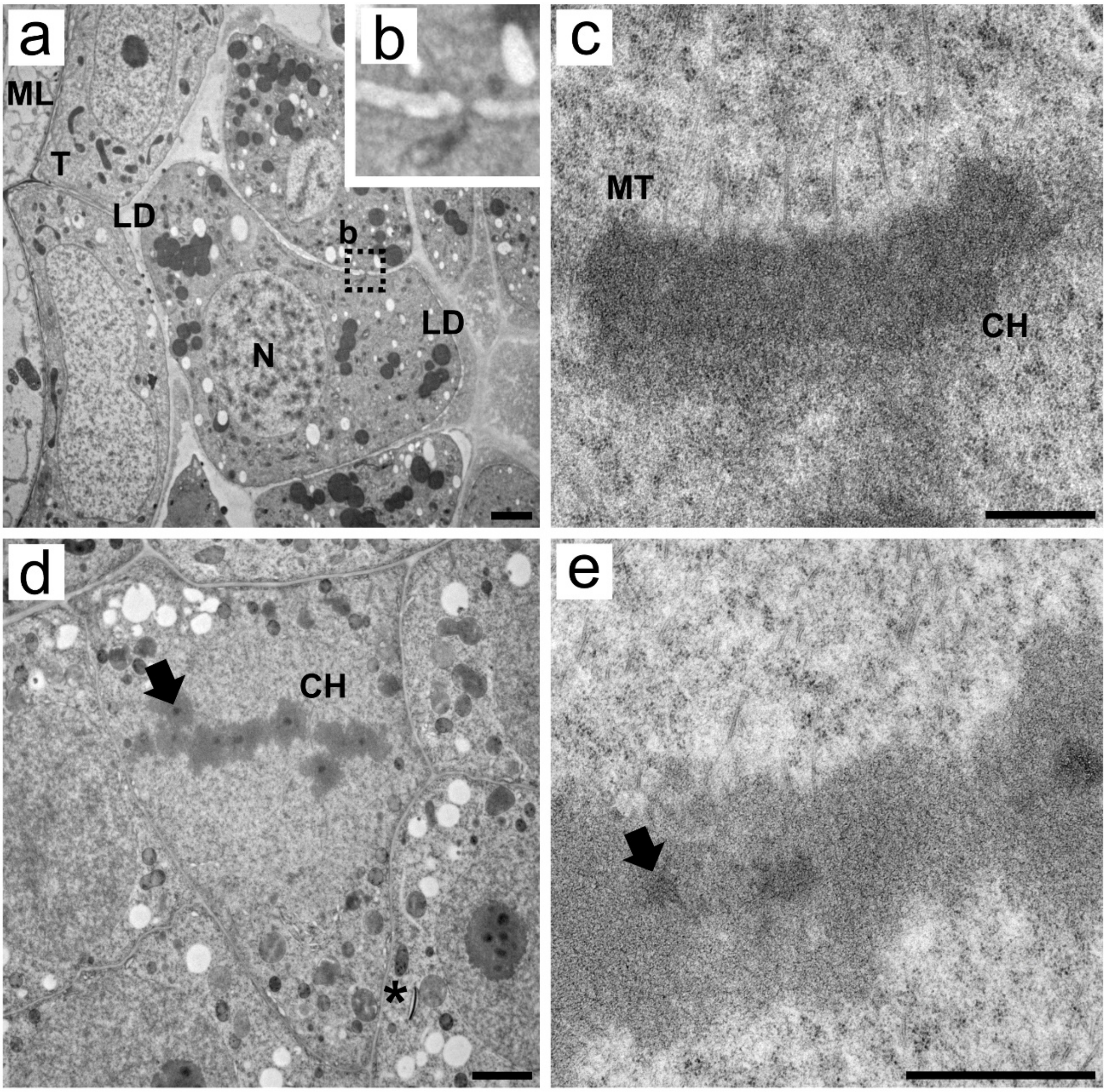

Figure S1. Transmission electron micrographs of ultrathin sections of different samples of *R. pubera* processed by chemical fixation. (a) Transversal section of an anther locule, showing middle layer (ML), tapetum (T) and an adjacent pre-meiotic microspore mother cell. The latter shows a prominent nucleus and organelles scattered throughout the cytoplasm, including electron-dense lipid droplets, as well as large cytoplasmic connections to adjacent microspore mother cells (**b**). Scale bar in **a** = 2 µm. (**c**) A mitotic chromosome (CH) in root cells, in a longitudinal section. This rod-shaped chromosome presents regular edges, where microtubules (MT) attach to. Scale bar = 500 nm. (**d**) A transversal section of the sporogenous tissue in a young anther shows mitotic chromosomes (CH) with electron-dense cores (Arrow). A rod-shaped electron-dense material is also seen in the cytoplasm (*). Scale bar = 2 µm. (**e**) Detail of the mitotic chromosome in (d) shows its regular edges and electron-dense central region. Scale bar = 500 nm.

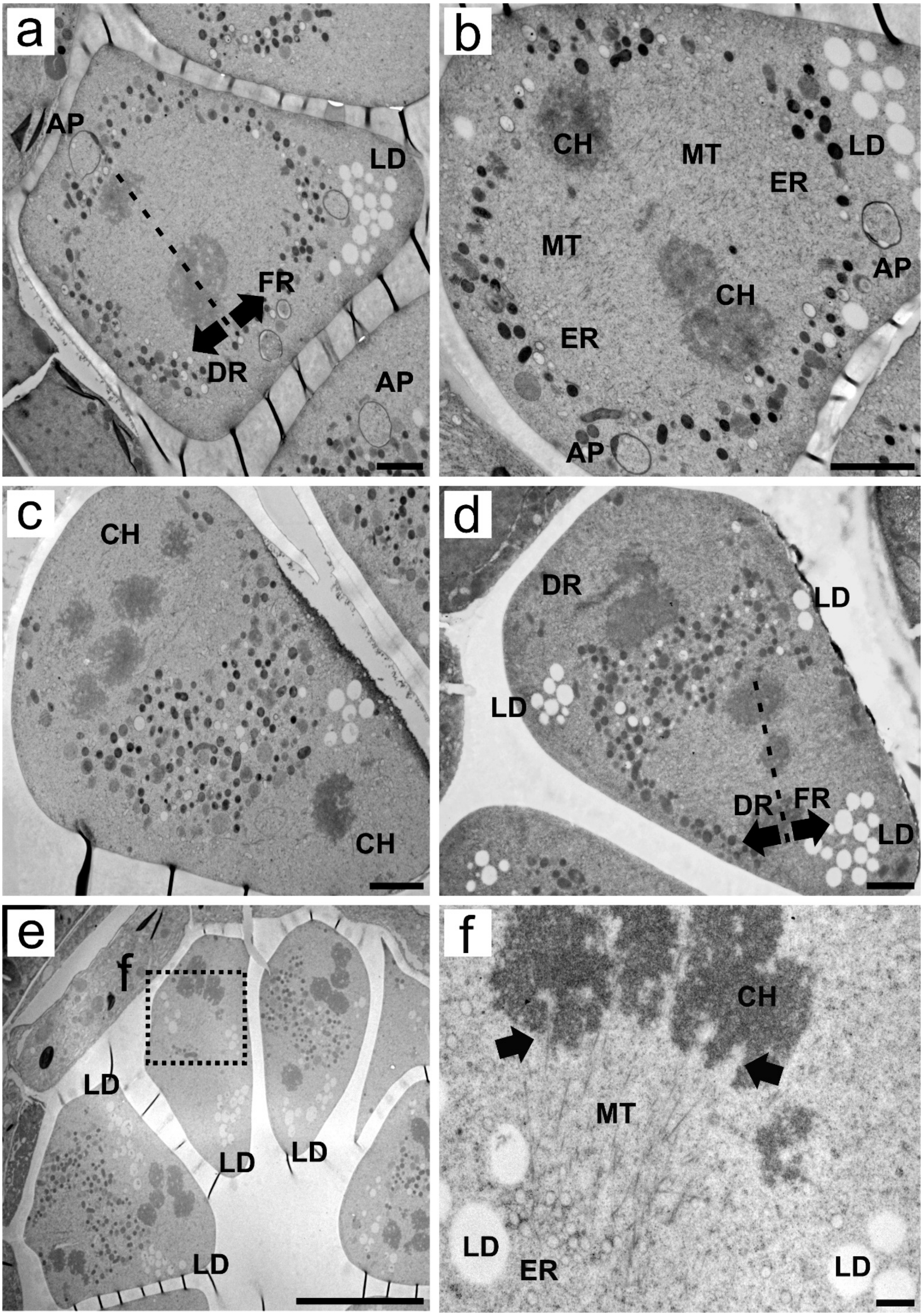
a
AP
LD
FR
DR
AP
b
CH
MT
LD
ER
MT
AP
CH
ER
AP
c
CH
CH
d
DR
LD
LD
DR
FR
LD
e
f
LD
LD
LD
LD
f
CH
MT
LD
ER
LD

Figure S2. Transmission electron micrographs of transversal ultrathin sections of microspore mother cells of *R. pubera* during meiosis. (a) At metaphase I, chromosomes are surrounded by a circular arrangement of organelles, which include autophagosomes (AP). Lipids droplets (LD) are located mainly in the apical region of the cell. Due to the tilted arrangement of the meiotic spindle, one of its poles faces the basal, degenerative region (DR) and the other faces the apical, functional region (FR). Scale bar = 2 µm. (**b**) During meiosis I, the spindle microtubules (MT) appear to be anchored by small endoplasmic reticulum cisternae (ER) at the spindle poles. Chromosomes (CH), autophagosomes (AP) and lipid droplets (LD) are also seen. Scale bar = 2 µm. (**c**) At prophase II, two chromosome sets (CH) are seen in opposite sides of the cell, with numerous organelles between them. Scale bar = 2 µm. (**d**) At metaphase II, the apical spindle presents one pole oriented towards the basal, degenerative region (DR) and the other to the apical, functional region (FR), adjacent to the apical lipid droplets (LD). These droplets are also seen in the angles of the cell. Scale bar = 2 µm. (**e**) Different cells in meiosis II are seen, with lipid droplets accumulating in their respective apical regions, as well as at their base angles. Scale bar = 10 µm. Micrograph of the boxed region in (**f**) shows that chromosome segregation in the basal region of the cell is also organized by spindle microtubules (MT) anchored to endoplasmic reticulum cisternae (ER) adjacent to lipid droplets (LD). The spindle microtubules penetrate deep inside the holocentric chromosome (arrows). Scale bar = 500nm.

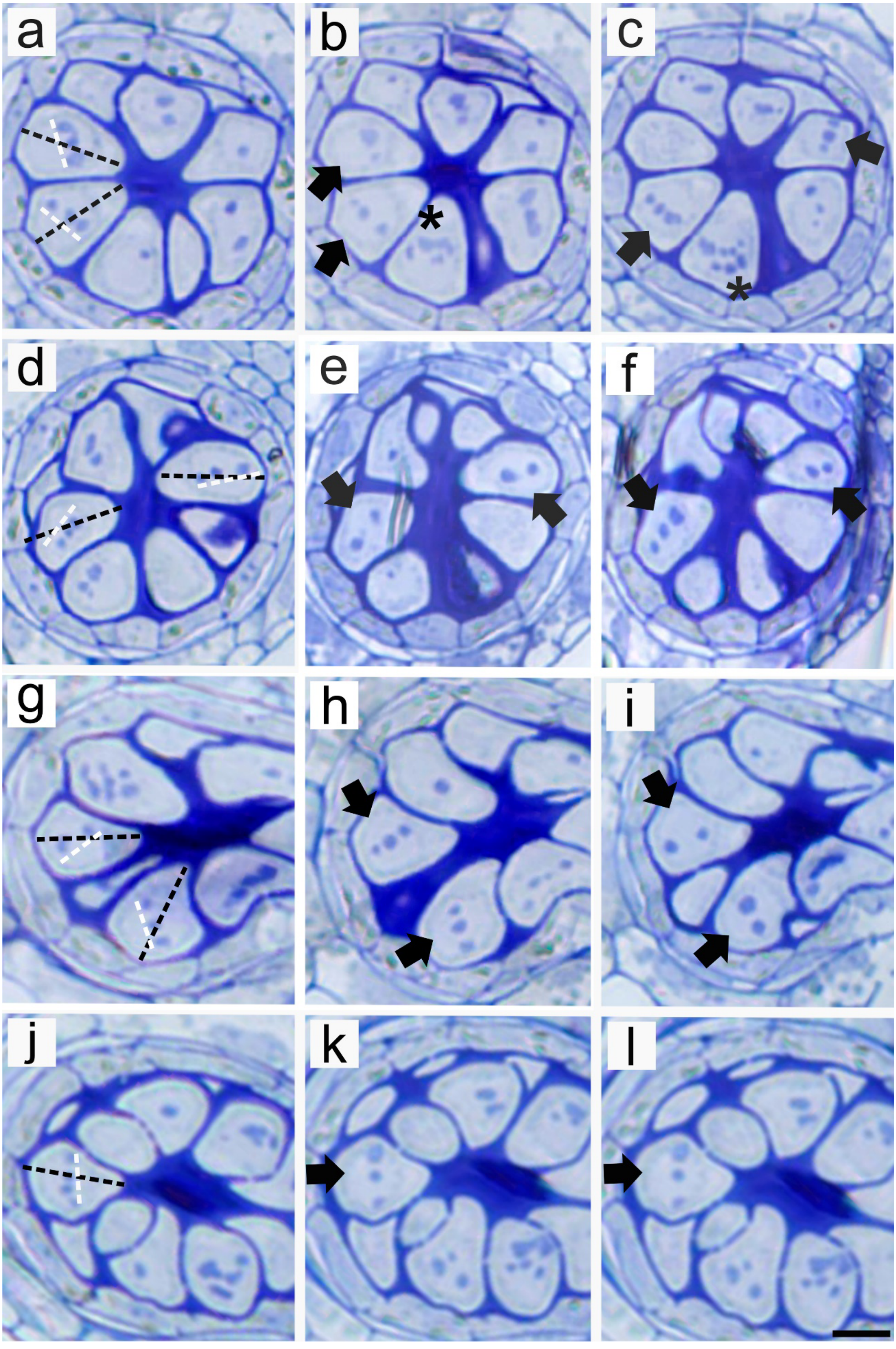
a
b
c
d
e
f
g
h
i
j
k
l

Figure S3. Light micrographs of serial transversal sections of an *R. pubera* anther in meiosis I. Three serial sections of the same locule are represented in **a-c**, **d-e**, **g-i** and **j-l**. In **a**, **d**, **g** and **j**, the orientation of the spindle (white dotted line) against the major axis of the cell (black dotted line) is represented. In the other images, the same cells are also seen in different sections (black arrows). Asterisks in **b** and **c** show two chromosome sets in different section planes, one to the left apical side and the other to the right basal side. Scale bar = 10 µm.

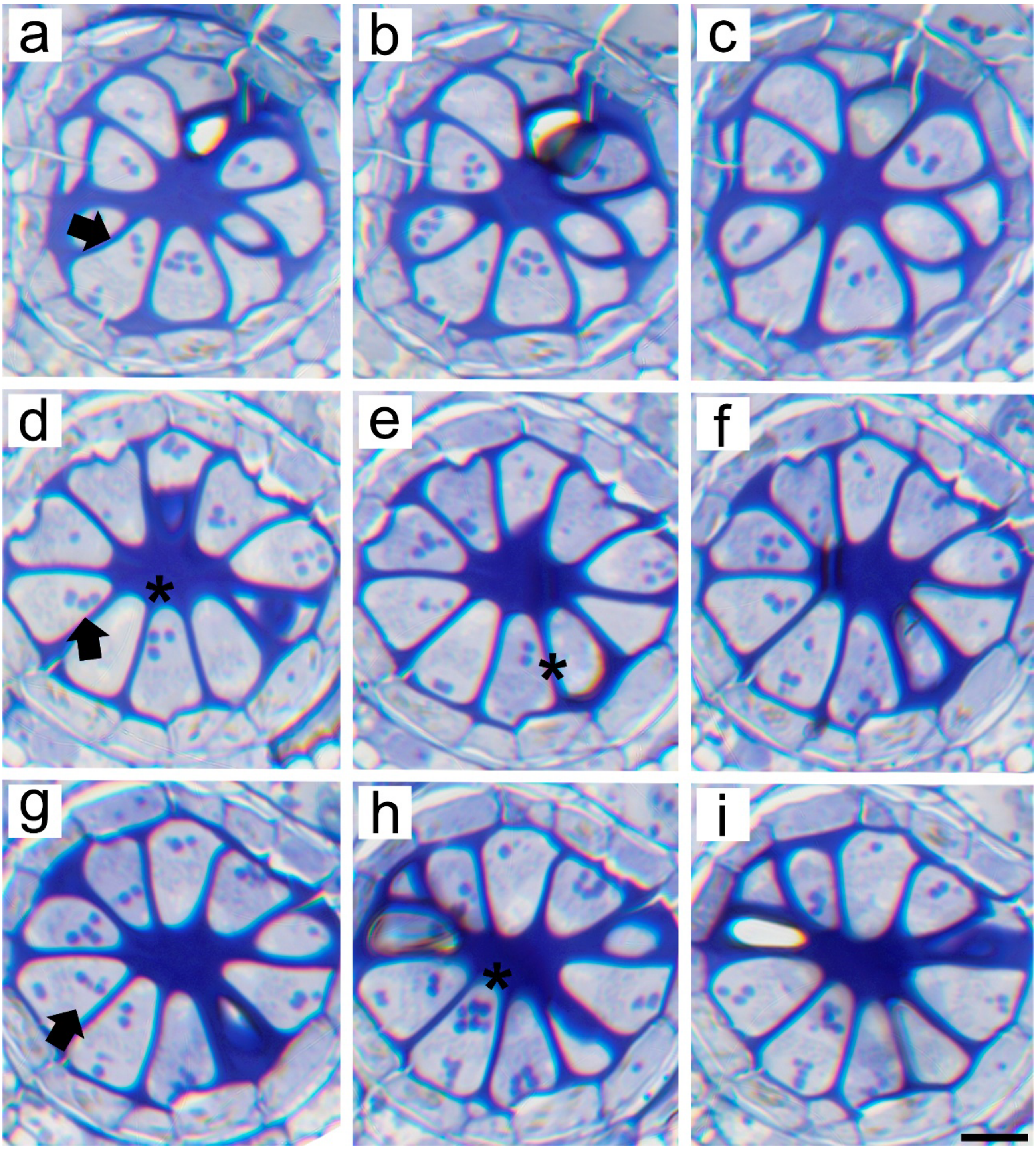

Figure S4. Light micrographs of serial transversal sections of an *R. pubera* anther in meiosis II. Three serial sections of the same locule are represented in **a-c**, **d-e** and **g-i**. Cells at metaphase II are seen with tilted spindles (black arrows). Scale bar = 10 µm. Asterisks in **d** and **e** show two chromosome sets in different section planes, one to the left apical side and the other to the right basal side. Figure **h** shows a possible anaphase II with the same configuration (*). Scale bar = 10 µm.

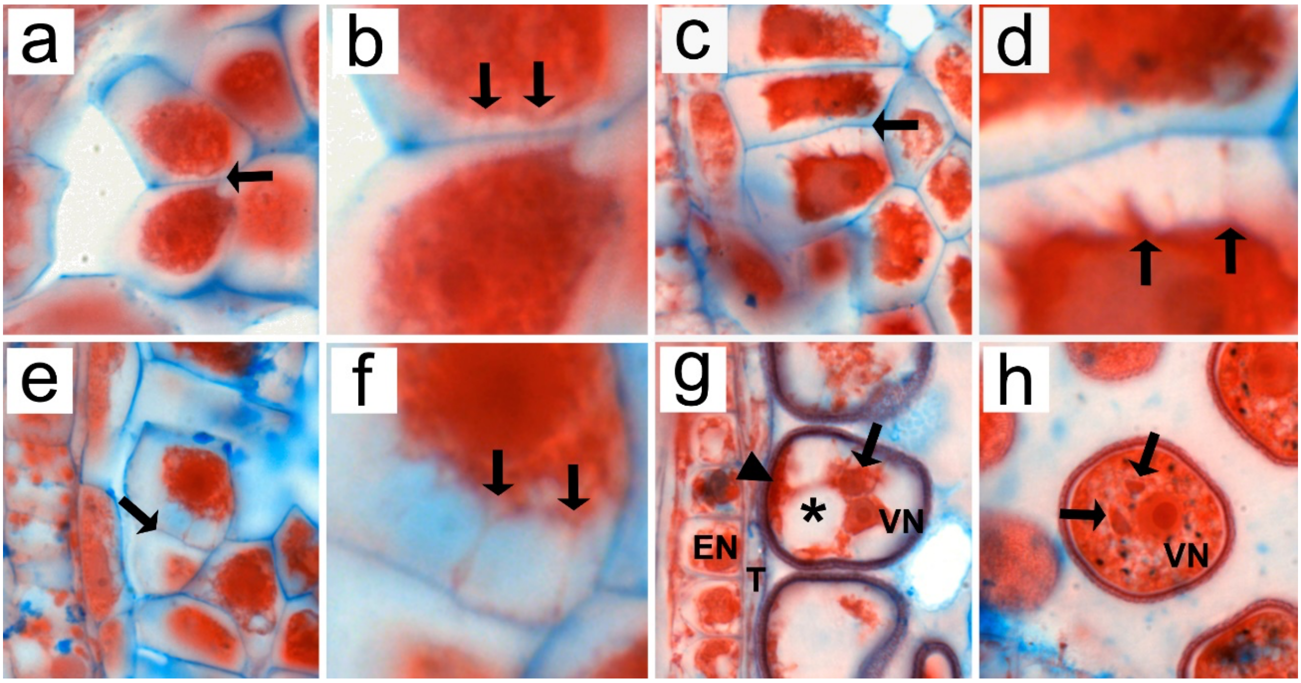

Figure S5. Light micrographs of longitudinal sections showing anther development in *R. pubera.* (**a**) Microspore mother cells present cytoplasm well stained by safranin, while pectic cell walls are stained by astra blue (arrow). Callose walls are unstained. (**b**) Detail of (a) showing cytoplasmic connections between adjacent microspore mother cells as thin threads crossing the common cell wall (arrows). The same is seen for other microspore mother cells (**c-d**, **e-f**). Scale bars in a, c and e = 10 µm. (g) After meiosis and pollen mitosis I, vegetative cells present large vacuoles (*). Remains of the degenerative nuclei are seen in the basal region of the pseudomonad (arrowhead). In the anther wall, the endothecium (EN) and remains of the tapetum are also seen (T). Scale bar = 10 µm. (**h**) Mature pollen grains of *R. pubera* present densely stained cytoplasm, with a vegetative cell, with a vegetative nucleus (VN), and two sperm cells adjacent to it (arrows). Scale bar = 10 µm.

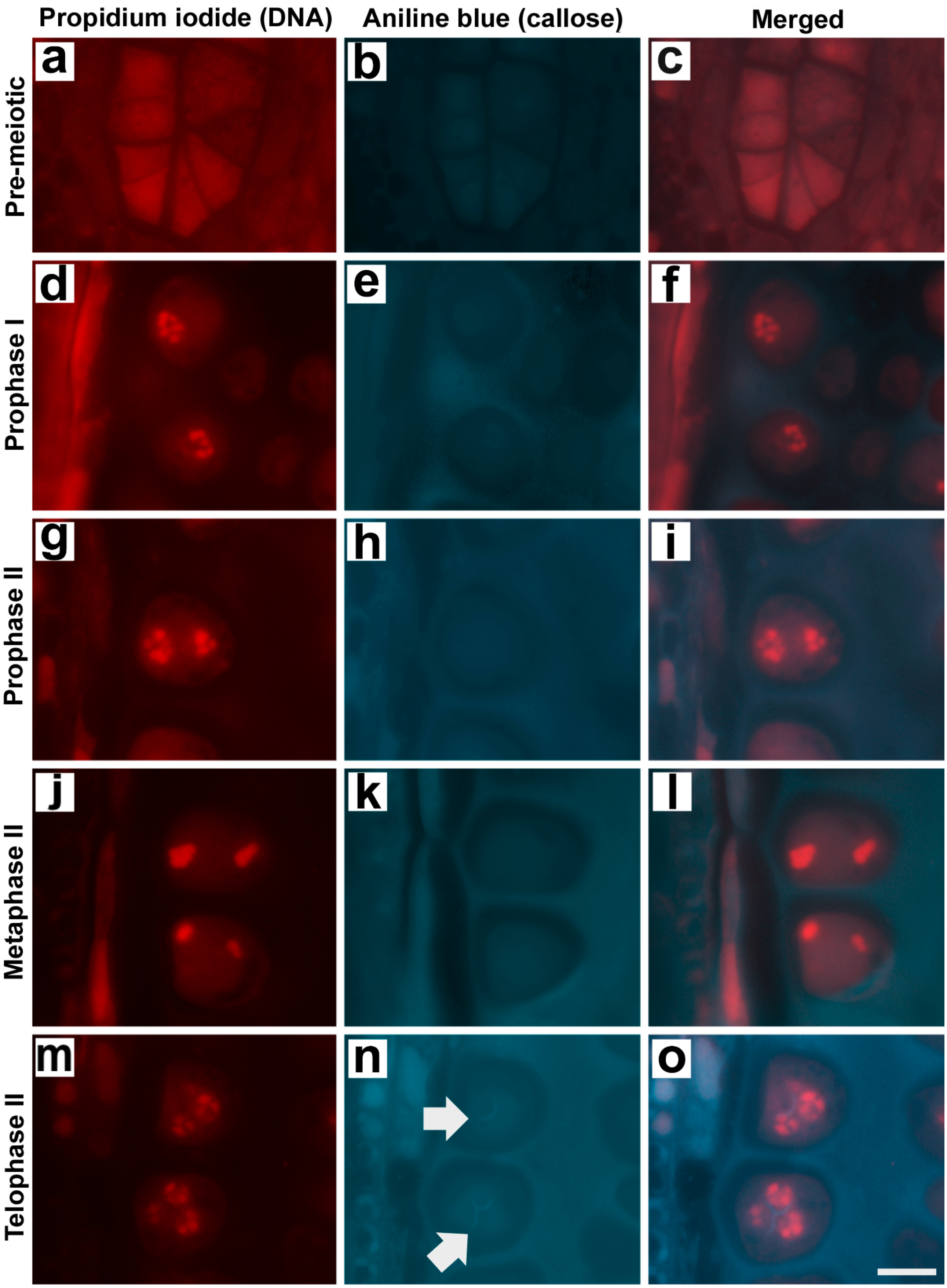
Propidium iodide (DNA)
Aniline blue (callose)
Merged
Pre-meiotic
Prophase I
Prophase II
Metaphase II
Telophase II
a
b
c
d
e
f
g
h
i
j
k
l
m
n
o

Figure S5. Epifluorescence micrographs of longitudinal sections of *R. pubera* anthers at different stages of development. Development series of MMCs from before meiosis to the establishment of asymmetrical tetrads following double staining with propidium iodide (**a, d, g, j, m**), Aniline Blue (**b, e, h, k, m**), which stains callose and merged images of propidium iodide and Aniline Blue (**c, f, i, l, o**). (a-c) Before meiosis, no callose deposition can be seen. (**d-f**) During prophase I, callose deposition becomes noticeable. Callose is still seen during prophase II (**g-i**) and metaphase II (**j-l**). At telophase II (**m-o**), callose is seen between microspore mother cells, as well as between the nuclei arising from meiosis (arrows in **n**). Scale bar = 10 μm.
